# Thinking eyes: visual thinking strategies and the social brain

**DOI:** 10.3389/fpsyg.2023.1222608

**Published:** 2023-09-27

**Authors:** Janneke E. P. van Leeuwen, Sebastian J. Crutch, Jason D. Warren

**Affiliations:** ^1^Dementia Research Centre, UCL Queen Square Institute of Neurology, University College London, London, United Kingdom; ^2^The Thinking Eye, ACAVA Limehouse Art Foundation, London, United Kingdom

**Keywords:** visual thinking strategies, VTS, art, images, social brain, artistic brain connectome, visual attention, eye-tracking

## Abstract

The foundation of art processes in the social brain can guide the scientific study of how human beings perceive and interact with their environment. Here, we applied the theoretical frameworks of the social and artistic brain connectomes to an eye-tracking paradigm with the aim to elucidate how different viewing conditions and social cues influence gaze patterns and personal resonance with artworks and complex imagery in healthy adults. We compared two viewing conditions that encourage personal or social perspective taking—modeled on the well-known Visual Thinking Strategies (VTS) method—to a viewing condition during which only contextual information about the image was provided. Our findings showed that the viewing conditions that used VTS techniques directed the gaze more toward highly salient social cues (Animate elements) in artworks and complex imagery, compared to when only contextual information was provided. We furthermore found that audio cues also directed visual attention, whereby listening to a personal reflection by another person (VTS) had a stronger effect than contextual information. However, we found no effect of viewing condition on the personal resonance with the artworks and complex images when taking the random effects of the image selection into account. Our study provides a neurobiological grounding of the VTS method in the social brain, revealing that this pedagogical method of engaging viewers with artworks measurably shapes people's visual exploration patterns. This is not only of relevance to (art) education but also has implications for art-based diagnostic and therapeutic applications.

## Introduction

The Swiss artist Paul Klee, whose seminal collection of notebooks on visual thinking inspired this study (Klee, 1961), famously said:

“Art does not represent the visible; rather, it makes visible.”

This insight does not only apply to the domain of the creative arts. It can also be extended to the scientific study of how human beings perceive and interact with their environment. We recently showed that art engagement and production recruit the same networks as complex social behavior (Van Leeuwen et al., 2022) by mapping neural correlates of visual art processing and visuospatial creativity onto the social brain connectome (Alcala-Lopez et al., 2017). The social brain connectome is a wiring diagram with functional profiles of the brain networks that guide social behavior. It was constructed from the most comprehensive quantitative meta-analysis of neuroimaging studies on social cognition to date and consists of thirty-six core social brain hubs spread across four hierarchical functional networks (Alcala-Lopez et al., 2017). The ‘artistic brain connectome' that we have based on the social brain connectome describes how the core social brain hubs and four networks are involved in art production and engagement (Van Leeuwen et al., 2022).

Building on the social brain connectome by Alcala-Lopez et al. (2017), we have described the four processing levels of the artistic brain connectome as follows (Van Leeuwen et al., 2022):

- Perceptual analysis (Perception Network). The process of coding complex visual phenomena, such as the ambiguity and incongruity in artworks and social scenes, is essential for understanding art and inter-personal interactions.
- Animating dynamics (Animation Network). Artworks, like people, require a creative orientation, whereby imagining and selecting potential responses are influenced by familiarity and emotional value. This processing is rapid and dynamic and entails novelty, making it integral to our subjective “aesthetic sense” when engaging with art.
- Interactive significance (Interaction Network). Viewer engagement with art is influenced by stored norms, perceived beauty, and personal homeostatic state. Analogous operations involve understanding and evaluating others' behaviors. Norm conformation and violation determine artwork salience, which is crucial for affective appraisal and artistic creativity.
- Symbolic and personal meaning (Construction Network). Art conveys mental states and the artist's intent, which requires interpretation to appreciate its meaning. Art mediates social cognition by constructing mental models of others' mental states, offering a window into the brain's internally constructed models of ourselves and others in relationship to the world around us.

We have proposed that this novel neuroscientific framework grounds art processes in the social brain and can guide further research in the field, as well as inform cultural and clinical applications. In this study, we applied this to an eye-tracking paradigm, which investigated how different viewing conditions and social cues influence gaze patterns and personal resonance with artworks and complex imagery in healthy young and older adults. We aimed to elucidate how viewing conditions that encourage personal and social perspective taking—modeled on the well-known Visual Thinking Strategies (VTS) method—might affect the visual exploration of social cues and personal resonance with artworks and complex imagery compared to settings in which only contextual information is provided. This is not only of interest with respect to (art) education but also has implications for art-based diagnostic and therapeutic applications.

Visual Thinking Strategies (VTS) is an art-based facilitated conversation method that engages people with visual artworks from their personal perspective in a social setting. As such, this method is ideally suited to explore the social brain dynamics of how people make sense of the social world, by studying how they interact with artworks. VTS was developed in the early 1990's in response to research that had shown that traditional gallery talks, in which an expert entertainingly explains the history and meaning of artworks, were not the most effective way for beginner art viewers to get a better appreciation for artworks that fell outside of what was already known and liked (Housen, 1987). Over the past decades, VTS has become internationally applied in an expanding range of educational, commercial, and therapeutic settings. The versatility of the method has been demonstrated by its ability to create accessible and inclusive art encounters in diverse settings, while nurturing careful observation, critical thinking, and collective meaning-making skills, as well as psychological and social wellbeing (Housen and Yenawine, 2000-2001; Housen, 2002; Yenawine, 2003; Naghshineh et al., 2008; Miller et al., 2013; Katz and Khoshbin, 2014; Van Leeuwen et al., 2021; Ferrara et al., 2022). The pedagogical principles of the VTS method have their roots in social constructivist theories (Bruner, 1960, 1986; Vygotsky, 1962). A core premise of social constructivism is that learning always takes place in a social context, and cognitive development is shaped through interactions with the environment and other people. This would suggest that VTS encourages the recruitment of social brain networks during encounters with artworks (and other complex imagery), which might underpin the cognitive and affective efficacy of VTS. Based on our knowledge of the social brain dynamics of art processing, we argue that when people look at artworks and complex images from a personal and social perspective (e.g., with the VTS method), this is likely to recruit the Construction Network of the artistic brain connectome to a higher degree than when people do not explicitly connect from a (inter-)personal angle. This network corresponds with the highest processing level in the social brain connectome and encompasses the Default Mode Network, which has been shown to play a key role in higher (social) cognitive functions, including language, and our knowledge of ourselves, others, and the world around us (Smallwood et al., 2021).

The social brain connectome provides a neural framework for linking perception to other mental processes and ultimately behavioral outputs when brains encounter complex social constructs such as artworks.

A functional neuroimaging study would be the most direct way to test this hypothesis, but functional neuroimaging techniques are difficult to translate to real-world environments and social contexts. Eye-tracking paradigms offer a non-invasive and flexible method to investigate how the brain processes information. Since the retina and visual pathway are part of the central nervous system, eye movements and gaze patterns provide a dynamic readout of brain activity (London et al., 2013). In addition, eye-tracking techniques are a potential bridge to more naturalistic environments via wearable technologies.

The scientific fascination with the eye as a mirror of the soul is demonstrated by the growing body of research that has investigated relationships between the visual exploration and aesthetic experience of artworks in both lay audiences and art experts (see for a review Rosenberg and Klein, 2015). Gaze patterns in real-world contexts are directed by an interplay between incoming information from the outside world and internal multi-modal knowledge systems and behavioral goals (Corbetta and Shulman, 2002; Henderson, 2003). Foveal vision (anatomically) only makes up the central 5° of the total human visual field, but it is responsible for a large amount of the visual information that reaches the visual cortex. This means that the eyes will constantly have to move around to perceive a visual scene sharply and in full color. A recent study reported that foveal vision anticipates the key features of fixation targets, which the authors identified as an important mechanism in the visual continuity of scene perception (Kroell and Rolfs, 2022). If perception is considered to be a form of hypothesis testing, whereby visual searches are aimed at optimizing information gathering (Friston et al., 2012), it could be argued that foveal vision leads to the most effective information gain. If the surface area of foveal vision during the exploration of a visual scene corresponds with the perceived salience of elements in that scene, it stands to reason these principles should also apply to visual artworks and complex images.

Previous research has found that a categorical distinction between “Animate” and “Inanimate” features is made very early in the human cortical visual processing system (Klein et al., 2009; Naselaris et al., 2012; Carlson et al., 2013; Proklova et al., 2016). “Anima” comes from the Latin “spirit/soul,” and the neuroscientific taxonomy of “animate” emphasizes the ability of self-movement (Ritchie et al., 2021), as well as the capacity to feel. The neuroscientific literature suggests that animate features in our environment carry the strongest social salience, and it has been shown that the mental representation of the social relevance of an external cue guides visual attention (Klein et al., 2009; Gobel et al., 2018). Visual attention in turn has been shown to enhance the strength of a stimulus by increasing its contextual contrast (Carrasco et al., 2004; Carrasco and Barbot, 2019). It has furthermore been shown that the simultaneous presentation of auditory and visual information also influences behavioral performance, whereby object familiarity and semantic congruency between the multisensory stimuli have facilitating effects (Laurienti et al., 2004; Hein et al., 2007; Ganczarek et al., 2022). However, the relationships between external information, visual attention, and internal mental states are not straightforward to decipher. Early eye-tracking studies reported that gaze patterns across photographs of a wide range of subjects (including artworks) varied depending on the task that people had been given (Buswell, 1935; Yarbus, 1967). Yet a more recent study failed to replicate Yarbus' findings, and the authors concluded that scan paths alone are not enough to decode the mental states of observers (Greene et al., 2012). The complex dynamics between internal states and engagement with the outer (social) world have been further demonstrated by studies that reported varying effects of personal preferences (Vessel et al., 2013; Herrera-Arcos et al., 2017), perspective taking (Beudt and Jacobsen, 2015), and different mental imagery styles (Felisberti and Cropper, 2023) on the aesthetic appreciation of photographs and visual artworks.

This complexity requires a relatively reductive experimental approach to deconstruct how external cues influence how people engage with visual artworks and complex imagery. For this reason, we designed a closely controlled eye-tracking paradigm that compared two viewing conditions that encouraged personal or social perspective taking—modeled on the VTS method—to a viewing condition during which only contextual information about the image was provided.

Based on our analysis of the existing literature, we hypothesized that (i) social cues (Animate vs. Inanimate) in artworks and complex imagery influence gaze patterns, (ii) audio cues that are presented during the exploration of artworks and complex imagery direct visual attention, (iii) viewing conditions that encourage personal or social perspective taking (VTS) direct the gaze more toward highly salient social cues (Animate elements) in artworks and complex imagery than providing contextual information, and (iv) viewing conditions that encourage personal or social perspective taking (VTS) lead to stronger personal resonance with artworks and complex imagery than providing contextual information.

## Materials and methods

### Participants

Seventeen young adults (Female *N* = 10) with a mean age of 27.7 (SD = 2.5) years and twenty older adults (Female *N* = 10) with a mean age of 64.4 years (SD = 8.3) were recruited via public social media, as well as via internal communication platforms at the Wellcome Collection and the UCL Dementia Research Center in London, where the study jointly took place. This study received ethics approval from the University College London Research Ethics Committee (8545/002: Created Out of Mind) and the UCL Queen Square Research Ethics Committee (17/LO/0099).

Participants had no history of neurological or visual disorders other than corrective lenses. A survey was conducted to collect data on the demographic background of the participants, including their experience with visual art (Table 1). A Wilcoxon paired samples test showed that the young and older adult cohorts only varied in age and not on any of the other demographic variables (*p* = 0.74). Baseline mood was measured with the Mood Shade Scale (Van Leeuwen, 2020), a novel 5-item visual rating scale that indicates mood states as spheres in different grades of lightness. The average self-reported mood of participants was bright, with a range between neutral and very bright (Supplementary Figure 1).

**Table 1:** Demographic background of the research participants. A Wilcoxon paired samples test showed that the young and older adult cohorts only varied in age and not on any of the other demographic variables (*p* = 0.74).

**Demographic background of research participants**		
**Research cohorts**	**Young adults**	**Older adults**
Total participants	17	20
Average age	27.7 (SD = 2.5)	64.4 (SD = 8.30)
**Personal characteristics ratios**		
Female	0.59	0.50
Male	0.41	0.50
Left-handed	0.05	0.20
Right-handed	0.95	0.80
UK-born	0.71	0.90
Born elsewhere	0.29	0.10
Ever lived abroad	0.59	0.65
**Highest education level ratios**		
GCSE/O levels	0.12	0
A-Levels	0.12	0.15
BA/BSc	0.35	0.3
MA/MSc	0.29	0.35
PhD	0.12	0.15
Unknown	0	0.05
**Art looking experience ratios**		
Hardly ever	0.06	0.10
A few times a year	0.18	0.20
Monthly	0.18	0.15
Weekly	0.29	0.15
Daily	0.24	0.35
Unknown	0.06	0.05
**Artistic style preference ratios**		
Figurative art preference	0.06	0.20
Abstract art preference	0.06	0.10
Both figurative and abstract art preference	0.88	0.60
Neither figurative nor abstract art preference	0	0.05
Unknown	0	0.05
**Art making experience ratios**		
Hardly ever	0.47	0.55
A few times a year	0.35	0.5
Monthly	0.12	0.10
Weekly	0.6	0.20
Daily	0	0.05
Unknown	0	0.05
**Creative art training experience ratios**		
Little to no training in creative art	0.35	0.060
Self-taught in creative art	0.24	0.10
Course(s) in creative art	0.24	0.25
Formal education in creative art	0.12	0
Unknown	0	0.05
**Art history/theory experience ratios**		
Little to no training in art history/theory	0.65	0.30
Self-taught in art history/theory	0.12	0.35
Course(s) in art history/theory	0.12	0.10
Formal education in art history/theory	0.12	0.10
Unknown	0	0.05

### Visual artworks and complex images stimuli

Thirty visual artworks and complex images from the Wellcome Collection were selected from their online and open-access image library (Supplementary Figure 2). The image selection included only figurative depictions of mostly public health and medical science-related subject matters, which is the core focus of the Wellcome Collection. The aim was to select a wide variety of image types and subjects to capture the natural dynamics of gaze dwell patterns across scenes with varying content of Animate and Inanimate image elements. The resulting image selection consisted of visual artworks and complex images in different media (drawing, painting, photography, and print), including both color and black and white images. All images were resized to a vertical dimension of 1,000 pixels and placed on a middle gray background (18%).

### Definition of foveal interest areas based on the social brain connectome

The thirty artworks and complex images that were used as stimuli in this study were parcellated into foveal interest areas (FIAs) using the SR Research Experiment Builder software. Each FIA had a diameter representative of the central visual field which can be perceived in color and high acuity by the human eye (5°). Building on research that showed that the categorization of “Animate” vs. “Inanimate” is made very early on during cortical visual processing (Klein et al., 2009; Naselaris et al., 2012; Carlson et al., 2013; Proklova et al., 2016), we divided the FIAs into two main categories: Animate and Inanimate image elements. Animate image elements were defined as “capable of self-movement” (Ritchie et al., 2021). Everything else was grouped under the Inanimate domain.

Within the Animate and Inanimate categories, subsets of foveal interest areas were created that were informed by a qualitative analysis of the functional profiles of core hubs in the social brain connectome (Alcala-Lopez et al., 2017). The authors of the social brain connectome calculated for a broad range of both social and non-social cognitive processes likelihood ratios in relation to the core social brain hubs, which gives an indication of which social brain areas and networks are likely to be involved in different social and non-social cognitive processes. We reasoned that the scale of these likelihood ratios could also reflect the significance (salience) of that particular cognitive process in the early stages of visual processing. Following this line of thought, the definition and rankings of the subcategories of foveal interest areas in this study were based on the average likelihood ratios (arrived at by forward inference) calculated across the six core nodes of the Perception Network in the social brain connectome (Alcala-Lopez et al., 2017). In the ranking order of the subcategories, we gave greater weight to functions with higher average likelihood ratios that recruited the highest number of core social brain hubs.

### FIAs in the Animate main category

Face monitoring had the highest average likelihood ratio (4.2) and engaged five out of the six core nodes in the Perception Network (Alcala-Lopez et al., 2017). Based on this, we reasoned that if any given visual scene contained facial features, these would have the highest social salience. Carlson et al. (2013) found that in the human cortical processing system of visual information, human faces formed a separate perceptual cluster after 120 ms, whereas monkey faces did not form into a distinct cluster until 180 ms after presentation. Based on this finding, we reasoned that human faces would be prioritized over animal faces. In addition to making a distinction between human and animal faces, separate perceptual categories were created for frontal and sideways faces. Warrington demonstrated that objects are better recognizable when they are observed from a standard (canonical) viewpoint (Warrington and Taylor, 1973). Based on this principle, we reasoned that frontal faces would be favored over sideways faces, reflected by longer average gaze dwell times. Action observation also recruited five out of the six core nodes in the Perception Network of the social brain connectome, with an average likelihood ratio of recruitment of 3.1 (Alcala-Lopez et al., 2017). Since hands are the most immediate and versatile tool that human beings have at their disposal for action execution, we created “Human hand actions” and “Animal hand actions” perceptual categories within the Animate domain, which were ranked directly below the face categories. As with the faces, we speculated that human hand actions would be more salient than animal hand actions during the visual exploration of a scene. The last two perceptual categories that were created within the Animate domain were for body parts other than faces and hands engaged in action. Again, it was reasoned that “Human body elements” would be more salient than “Animal body elements,” and the first was therefore ranked higher.

### FIAs in the Inanimate main category

“Text elements” were ranked the highest in the Inanimate domain, based on the fact that semantic monitoring/discrimination recruited five of six core hubs of the Perception Network in the social brain connectome, with an average likelihood ratio of recruitment of 3.1 (Alcala-Lopez et al., 2017). Visual object recognition engaged just one of the six core nodes in the Perception Network of the social brain connectome, but it had a very high likelihood ratio of recruitment (11). Therefore, the category “Human-made objects” was ranked directly under “Text elements” in the Inanimate domain. We created a separate perceptual category for “Built environment elements,” which was ranked under “Human-made objects” in the Inanimate domain. Natural elements were not represented in the social brain connectome, but it is a highly relevant perceptual category in the context of the visual exploration of visual art and complex imagery. Since we had no likelihood ratios from the social brain connectome as a guidance for this category, we ranked “Natural elements” under “Built environment elements,” reasoning that man-made elements would have stronger social salience than natural elements. Counting/calculation recruited four out of the six core nodes in the Perception Network of the social brain connectome, with an average likelihood ratio of recruitment of 1.0. The final perceptual category of the Inanimate domain was therefore “Number elements.”

Supplementary Table 2 shows the ranking of the different subsets of FIAs that were created within the Animate and Inanimate main categories, accompanied by average gaze dwell times across the three different viewing conditions. The dwell-time distribution across the different FIA subsets largely aligned with our proposed ranking order, which supports the rule system we created for the allocation of fixations to distinct perceptual categories (further detailed in the section below).

### Placement of FIAs on the artworks and complex images

The center coordinates of each FIA were determined by drawing a rectangular outline around an image feature and then aligning the center of the circular FIA with the center of the rectangular outline. The FIA center coordinates were defined as the most efficient gaze orientation, and fixations within a 2.5° visual angle around the center coordinates were considered to result in the most optimal visual information gain. The diameter for the FIAs was calculated with the use of the Pythagorean theorem by multiplying the tangent function of 2.5° with 75, the distance in cm between the participant's eyes and the image display. The resulting circle radius of 3.3 cm was converted to pixel dimensions, using a cm-to-pixel ratio of 1:37.795 (https://www.unitconverters.net), which corresponded to a digital circle diameter of 250 pixels.

The motivation to generate circular interest areas that aligned with the circle area of the foveal vision, rather than interest areas based on feature outlines (or more abstract even; image pixel resolutions), was that this method is more closely aligned with the temporal dynamics and physiological restrictions of visual processing. After all, what the brain can process in color and with high acuity is not primarily defined by the features of a visual scene, but by whatever visual information falls within the perimeter of the foveal visual field.

Diverse image features of the same feature category that were too large to be captured by a single FIA were divided up into separate FIAs that covered the different aspects, whereby lighter/brighter colored and nearer features were prioritized. If two or more separate features belonging to the same feature category could be fixated with a single FIA, the rectangular outline was drawn around the grouped feature surface. Different facial features were categorized under either the frontal or the sideway facial category, depending on the orientation of the face. Faces that were turned only slightly sideways, with nose, mouth, and both eyes clearly visible, were assigned to the “Face frontal” category if the gaze was directed toward the viewer and assigned to the “Face sideways” category if the eyes were not directed toward the viewer. Elements attached to a face were included in the feature outline rectangle, as were the elements that were held in the hand-action categories. FIAs that covered human or animal body elements that did not contain visible faces or hands and were largely or entirely covered by clothing or other covering material were assigned to the “Human-made objects” category, rather than the “Human/animal body element” category. Different image features that had overlapping FIAs were only designated separate interest areas if their centers were more than 125 pixels (the radius of the foveal visual field) apart from each other. If the distance was equal to or less than 125 pixels, a single foveal fixation area was placed on the center of the feature highest up in the proposed visual processing hierarchy. Fixations that fell in overlapping FIAs were automatically allocated to the higher-ranked FIA by the processing software (SR Research Data Viewer). Image elements that were identifiably referenced in an audio recording that was played simultaneously with the image presentation were allocated an FIA with an audio marker.

Table 2 details the distribution of foveal interest areas (FIAs) with and without audio markers across the artworks and complex images that were shown under the three different viewing conditions: contextual information, external perspective (VTS), and internal perspective (VTS).

**Table 2:** Distribution of foveal interest areas (FIAs) with and without audio markers across the artworks and complex images that were shown under the three different viewing conditions: contextual information, External perspective, and internal perspective.

**Viewing condition**	**Image**	**Animate FIAs with audio marker**	**Inanimate FIAs with audio marker**	**Animate FIAs without audio marker**	**Inanimate FIAs without audio marker**
Contextual information	1	6	0	4	2
Contextual information	3	4	0	3	9
Contextual information	4	1	0	5	12
Contextual Information	7	2	1	10	5
Contextual information	9	0	7	0	2
Contextual information	11	2	3	0	2
Contextual information	13	10	0	1	0
Contextual information	15	2	0	3	9
Contextual information	17	1	4	0	11
Contextual information	19	1	0	0	10
**Contextual information**	**Average**	**29**	**15**	**26**	**62**
External perspective	2	0	1	9	3
External perspective	5	2	2	1	2
External perspective	6	0	2	3	4
External perspective	8	1	0	7	6
External perspective	10	5	0	0	9
External Perspective	12	1	0	7	13
External perspective	14	0	1	0	22
External perspective	16	0	1	0	4
External Perspective	18	2	1	9	8
External perspective	20	0	2	7	5
**External perspective**	**Average**	**11**	**10**	**43**	**76**
Internal perspective	21	0	0	5	12
Internal perspective	22	0	0	6	6
Internal perspective	23	0	0	4	2
Internal perspective	24	0	0	3	2
Internal perspective	25	0	0	2	8
Internal perspective	26	0	0	2	8
Internal perspective	27	0	0	4	15
Internal perspective	28	0	0	7	5
Internal perspective	29	0	0	12	9
Internal perspective	30	0	0	5	8
Internal perspective	Average	0	0	50	75

**Figure 1:**
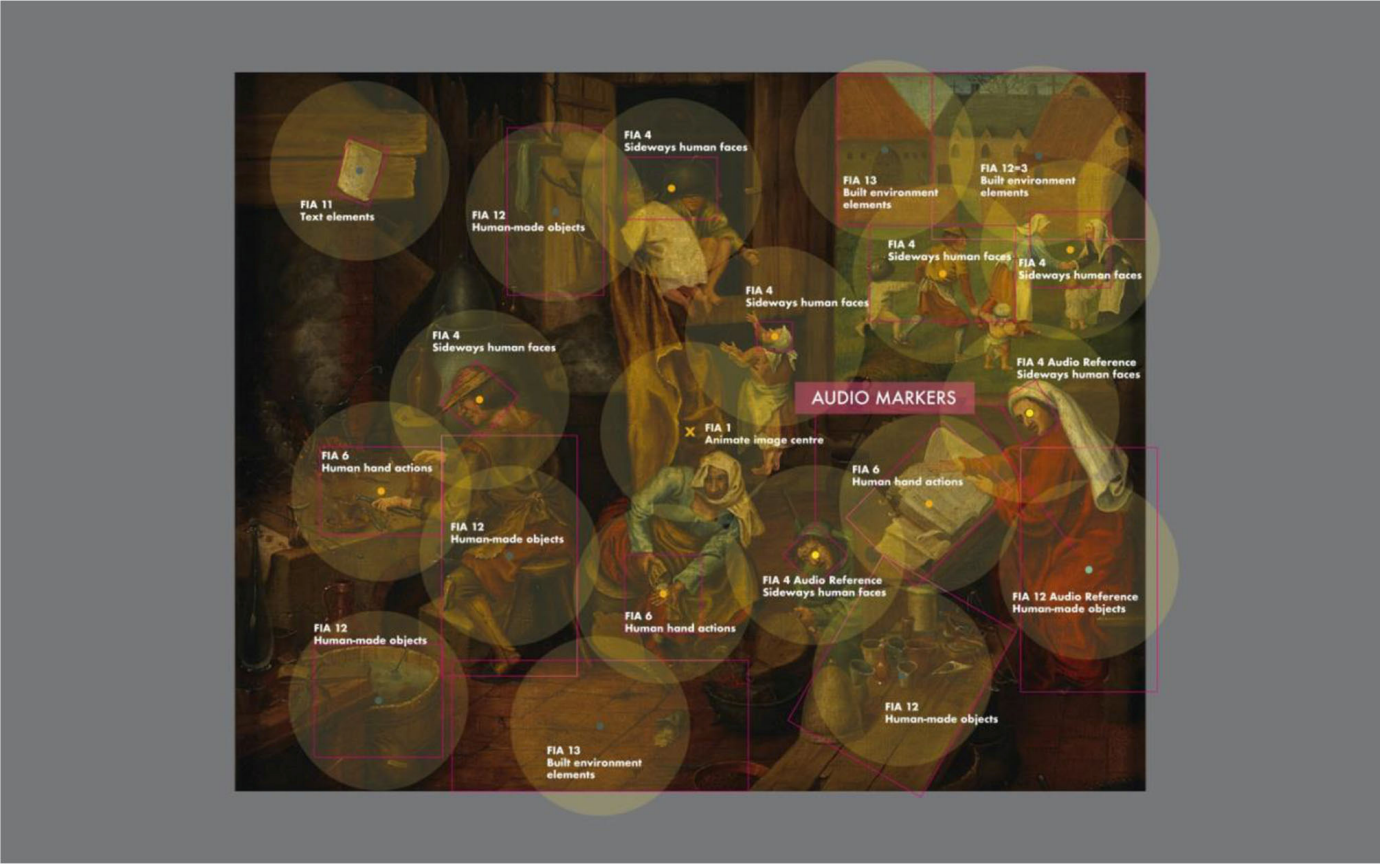
Illustration of the placement of foveal interest areas (FIAs) on the visual artworks and complex imagery used as experimental stimuli. The color coding of the central points of the FIAs is aligned with the two main categories: Animate (orange) and Inanimate (dark green). Separate color coding was applied to Animate FIAs with audio markers (yellow) and Inanimate FIAs with audio markers (light green). The shown example is image #18 (image attribution under Supplementary Table 1, image number 18).

Figure 1 illustrates the placement of FIAs across one of the artworks that were used as a visual stimulus in this study.

### Content of auditory stimuli for the different viewing conditions

There were three different viewing conditions, which each consisted of 10 different images from the selection of 30 figurative visual artworks and complex images from the Wellcome Collection in London. Each image was presented for 20 s, while an audio recording was played as well during part of the image presentation. All the audio stimuli were recordings of the same female voice—a study volunteer with native British nationality and an excellent command of Standard British English who was not involved in the research development.

In the first viewing condition “Contextual information,” participants listened to contextual information that the Wellcome Collection had provided about that image in their online catalog. This was typically the kind of information that is usually written on a wall label next to an artwork in a museum (e.g., a content description, name of the artist, and year of production). The External perspective viewing condition was modeled on the social scaffolding aspect of the VTS method, whereby participants are exposed to other people's perspectives during a collective exploration of an artwork or complex image. The audio stimuli in the External perspective viewing conditions consisted of a fragment of the study volunteer's unscripted response to the standard first question each VTS conversation opens with “What is going on in this picture?”. The audio files from the External perspective viewing condition were edited down to only include the first couple of sentences of the personal reflection, to ensure the cognitive load of these audio stimuli was not much larger compared to the Contextual information viewing condition. In the contextual information viewing condition, there were 29 Animate FIAs with audio markers and 15 Inanimate FIAs with audio markers, compared to 11 Animate FIAs with audio markers and 10 Inanimate FIAs with audio markers in the External perspective viewing condition. Ideally, the numbers of FIAs with audio markers would have been perfectly matched between these two viewing conditions, but given the unscripted nature of the audio recordings in the External perspective viewing condition, this was difficult to achieve. We controlled for any random variation caused by the distribution of FIAs across the individual images in the statistical analysis, however. In the third viewing condition “Internal perspective,” the same audio stimulus was played during every image trial and consisted of the VTS prompting question: “What is going on in this picture?”. Participants had been instructed at the start of the experiment to reflect on this question internally and not out loud.

Supplementary Table 3 shows the transcripts of the audio stimuli that were played to the research participants during the presentation of the artworks and complex images in the Contextual information and External perspective (VTS) viewing conditions.

### Apparatus

The eye-tracking experiment was programmed and run using the SR Research Experiment Builder software package. The 30 visual artworks and complex imagery stimuli were presented on an Eizo ColorEdge CG2420 24-inch LCD monitor, which was placed at a 75 cm distance from a table-mounted headrest that stabilized the chin and forehead of the participants. For each participant, the height of the chinrest was adjusted so that their eyes aligned with the top 25% of the monitor. The display area of the monitor measured 518.4 x 324.0 mm with a native resolution of 1920 x 1200 (16:10 aspect ratio) and was calibrated with an X-rite Eye One Display 2 device, using Eizo ColorNavigator6 software which was installed on a connected 13-inch late 2016 Macbook Pro laptop from which the experiment was run. The target color profile of the Eizo ColorEdge CG2420 24-inch LCD monitor was defined within the sRGB color space at a brightness of 100 cd/m^2^, a white point of 6,500 K, and the brightness level of black set to 0.5 cd/m^2^. The tone curve of the monitor was defined at an RGB gamma of 2.2 with a standard priority. The monitor background color behind each stimulus presentation was middle gray (18%). An SR Research Eyelink 1,000 Plus eye-tracking camera was placed in front of the monitor at a 55 cm distance from the headrest, with the lens directed at the eyes of the participant. The eye-tracking camera was calibrated to each individual participant with the Experiment Builder software, using a 9-point grid. Bilateral fixations were recorded at a frequency of 1,000Hz, but only the fixation data of the right eye recordings were used in the data analysis. The SR eye-tracking software identified the pupil outlines based on the darkest area within the recording grid of the eye-tracking camera, which was determined by the refraction pattern of an unobtrusive infrared beam that was emitted by the eye-tracking camera. The eye-tracking data were recorded onto a Dell laptop, which was connected to the Macbook Pro laptop that ran the experiment. Both laptops were placed on a black table which was positioned at a 90° angle to the left of the experiment presentation table. Supplementary Figure 3 shows the eye-tracking experiment setup (during the experiment the ambient light was turned off).

### Procedure

The eye-tracking experiment took place in a blackout room. Research participants were dark-adapted for a minimum of 10 min. Before the experiment started, a pre-recorded audio instruction explained the procedure of the experiment and participants were given the opportunity to ask questions if anything was unclear.

Each image trial began with a 3 s presentation of a middle gray (18%) screen (1,574 x 1,050 pixels at 96 dpi) with a fixation cross in the middle to (re)orientate the gaze toward the center of the screen. Participants were then shown 30 visual artworks and complex images in 3 blocks of 10 different images, which were presented for a duration of 20 s each. As people were looking at the visual artworks and complex images, they also listened to an audio recording, the style and content of which varied across the three different viewing conditions. In the first two blocks, the “contextual information” and “External perspective” viewing conditions were alternated in a pseudo-randomized manner, followed by the “internal perspective” block. The rationale for this design was to avoid the risk that participants would automatically ask themselves the question “What is going on in this picture?” in all three viewing conditions, once they had been asked this question once.

After each image presentation, participants were asked to say out loud how much it resonated with them using the Resonance Radius Scale, a novel visual rating scale specifically designed for this study (Supplementary Figure 4).

### Gaze data processing

The recorded eye fixation data were pre-processed with the SR Research Data Viewer software. Blinks were filtered out in Data Viewer, based on the occurrence of small data gaps in the continuous recordings of fixations, and only the fixation recordings between the 750- and 20,000-ms interval after presentation in the eye-tracking experiment were exported for the data analysis. The rationale behind the choice of this data sampling window was that this would allow us to analyze the gaze patterns that aligned with higher-order cortical processing phases (Cela-Conde et al., 2013; Van Leeuwen et al., 2022). The gaze data were exported by Data Viewer as average dwell times on individual FIAs per image per participant.

### Statistical analyses

All statistical analyses were carried out using the JASP statistical software (JASP TEAM, 2023), version 0.17.1. Three mixed models were fitted on the dataset to analyze the effects of different viewing conditions, audio input, and social cues on gaze patterns and personal resonance with visual artworks and complex imagery in healthy young and older adults. The average gaze dwell times (ms) on the individual FIAs in each image were averaged across the Animate and Inanimate categories and split into with/without audio marker subcategories. Two linear mixed models (LMMs) were fitted to the gaze data using the Satterthwaite method, a restricted maximum likelihood test that has field degrees of freedom across tests. This is the most appropriate method when the sample size is small, or the data are unbalanced, which was the case in this study. For the (ordinal) personal resonance data, a generalized linear mixed model (GLMM) was fitted using the Gaussian family and link identity function. For all analyses, a *p*-value below 0.05 was considered as a statistically significant effect. In addition to p-values, the Vovk-Sellke maximum p-ratio (VS-MPR) was calculated for each effect. The VS-MPR functions as a Bayes factor and describes how the data have changed the likelihood of the alternative hypothesis compared to the null hypothesis. When the alternative hypothesis H1 is true, small *p*-values are more likely to occur than large p-values. When the true effect is modest however, small *p*-values are only a little more likely than large p-values. When the true effect is significant, small *p*-values are much more likely than large p-values. This difference in the likelihood of obtaining small *p*-values when the true effect is modest or significant is addressed by the VS-MPR, which gives an indication of the so-called diagnosticity of a two-sided p-value. The VS-MPR shows the maximum odds in favor of the alternative hypothesis (H1) over the null hypothesis (H0), defined as 1/(-e p log(p)) for *p* ≤ 0.37 (Sellke et al., 2001). Using Bayes factors in combination with p-values has been shown to mitigate the risk of Type 1 errors (Benjamin and Berger, 2019).

Model 1: A linear mixed model was fitted to the average dwell times on Animate and Inanimate foveal interest areas (FIAs)—without audio markers—under the three different viewing conditions (Contextual information, External perspective, and Internal perspective). Fixed effects were calculated for “Viewing condition” with 3 levels and “FIA category” with 2 levels (Animate, Inanimate), with “Image” (*N* = 30) selected as a random effects grouping factor. The LMM random-effect structures for “Image” included the intercept and the random slopes for the factor “FIA category.” All random slopes involving “Viewing condition” were automatically removed from the model by JASP, as the factor ‘Viewing condition' did not vary within the levels of random effects grouping factor ‘Image'. The dwell-time data that were included in the analysis were recorded between 750 and 20,000 ms after image presentation, reflecting the time window during which higher cortical processes are recruited in the image processing (Cela-Conde et al., 2013; Van Leeuwen et al., 2022). Estimated marginal means were calculated for the two FIA categories across the three viewing conditions.

Model 2: A linear mixed model was fitted to the average dwell times on Animate and Inanimate foveal interest areas (FIAs) with and without audio markers under the two viewing conditions that involved audio stimuli that referenced visual elements in the artworks and complex images: Contextual information and External perspective. Fixed effects were calculated for “Viewing condition” with 2 levels and “FIA subcategory” with 4 levels (Animate, Inanimate, Animate audio marker, and Inanimate audio marker), with “Image” (*N* = 20) selected as a random effects grouping factor. A random intercept for “Image” was added to the LMM. The dwell-time data that were included in the analysis were recorded between 750 and 20,000 ms after image presentation. Estimated marginal means were calculated for the four FIA subcategories across the two viewing conditions.

Model 3: A generalized linear mixed model was fitted to the personal resonance ratings of the visual artworks and complex images (*N* = 30), with “Viewing condition” as a fixed effect and “Image” (*N* = 30) as a random effects grouping factor.

Estimated marginal means were calculated for the resonance ratings across the three different viewing conditions (Contextual information, External perspective, and Internal perspective). The personal resonance ratings from one participant in the young adults group were excluded from the analysis because their ratings were much lower than the cohort averages across all experimental conditions (>2 std dev), possibly suggestive of an indiscriminate low engagement with and/or dislike of the image selection.

## Results

None of the three mixed models showed main effects of the factors “Cohort” and “Sex”, indicating that neither age nor sex influenced gaze patterns and personal resonance with visual artworks and complex imagery in this study. Reflecting this, the reported results here concern the averaged responses across all research participants.

Model 1 (Table 3) found strong evidence for a main effect of the factor “FIA category,” indicating that social cues (Animate vs. Inanimate) had an influence on gaze patterns across visual artworks and complex images. Some evidence was found for a main effect of “Viewing condition,” but there was stronger evidence for an interaction between “Viewing condition” and “FIA category,” suggesting that the three different viewing conditions had specific effects on the visual attention for social cues in the artworks and complex images. The largest difference in gaze patterns was observed between the Internal perspective (VTS) and the Contextual information viewing conditions. The average time that participants spent looking on Animate image elements in the Internal perspective (VTS) viewing condition was estimated to be 2,662 ms ± 275 ms (SE), which was 3.5 times longer compared to the estimated average of 754 ms ± 350 ms (SE) in the Contextual information viewing condition (*p* = 0.001, VS-MPR = 40.980). The estimated average dwell time on Animate image elements in the External perspective (VTS) viewing condition was 1,627 ms ± 323 ms (SE). The estimated average dwell times on Inanimate image elements in the External perspective and Internal perspective (VTS) viewing conditions were 585 ms ± 175 ms (SE) and 687 ms ± 175 ms (SE), respectively. This was comparable to the estimated average dwell time on Inanimate image elements in the Contextual information viewing condition, 778 ms ± 184 ms (SE). However, in both the external and the internal (VTS) viewing conditions, the estimated average dwell times on Animate image elements were significantly longer than on Inanimate image elements, whereas, in the Contextual information viewing condition, the estimated average dwell times on Animate and Inanimate image elements were almost the same.

**Table 3:** Linear mixed model (Model 1) was fitted to the average dwell times on Animate and Inanimate foveal interest areas (FIAs)—without audio markers—under the three different Viewing conditions (contextual information, External perspective, and internal perspective).

**Model 1: Effects of different viewing conditions and social cues on gaze patterns across visual artworks and complex images in healthy adults**						
**Effect**			**df**	**ChiSq**	***p***	**VS-MPR** ^*^
Viewing condition			2, 21.68	5.453	0.012	6.899
Foveal interest area (FIA) category			1, 20.88	28.741	< 0.001	1333.123
Viewing condition * FIA category			2, 20.66	9.820	0.001	53.007
**Fixed effect estimates**						
**Term**	**Estimate**	**SE**	**df**	***t***	***p***	**VS-MPR** ^*^
(Intercept)	1182.309	115.855	21.940	10.205	< 0.001	2.055 × 10^+7^
**Reference viewing condition: internal perspective**						
Viewing condition: contextual information	−416.025	169.408	23.271	−2.456	**0.022**	**4.391**
Viewing condition: xternal perspective	−76.324	164.697	21.930	−0.463	0.648	1.000
**Reference FIA category: inanimate**						
FIA category: animate	498.940	93.068	20.884	5.361	**< 0.001**	**1333.125**
**Interaction effects**						
Animate FIAs * Contextual information viewing condition	−510.802	139.196	21.809	−3.670	**0.001**	**40.980**
Animate FIAs * External perspective viewing condition	22.479	132.343	20.511	0.170	0.867	1.000
**Estimated marginal means**						
						95% CI
Viewing Condition		Foveal interest area subcategory	Estimate	SE	Lower	Upper
Contextual information		Animate	754.423	349.593	69.233	1439.612
External perspective		Animate	1627.405	323.435	993.483	2261.326
Internal perspective		Animate	2661.922	274.834	2123.258	3200.586
Contextual information		Inanimate	778.146	183.653	418.192	1138.099
External perspective		Inanimate	584.566	175.287	241.010	928.122
Internal perspective		Inanimate	687.395	175.208	343.993	1030.797

Model terms tested with the Satterthwaite test method.

The following variable is used as a random effects grouping factor: “Image”.

Type III sum of squares.

^*^Vovk-Sellke maximum p-ratio: Based on a two-sided p-value, the maximum possible odds in favor of H1 over H0 equal 1/(-e p log(p)) for p ≤ 0.37 (Sellke et al., 2001).

The intercept corresponds to the (unweighted) grand mean; for each factor with k levels, k - 1 parameters are estimated with sum contrast coding. Consequently, the estimates cannot be directly mapped to factor levels. The estimated marginal means below provide estimates for each factor level/design cell and their differences.

Fixed effects were calculated for the “viewing condition” with 3 levels and the “FIA category” with 2 levels (Animate and Inanimate), with ‘Image' (*N* = 20) selected as a random effects grouping factor. The LMM random-effect structures for “Image” included the intercept and the random slopes for the factor “FIA category.” All random slopes involving “viewing condition” were automatically removed from the model by JASP, as the factor “Viewing condition” did not vary within the levels of random effects grouping factor “Image.” The dwell-time data that were included in the analysis were recorded between 750 and 20,000 ms after image presentation, reflecting the time window during which higher cortical processes are recruited in the image processing (Cela-Conde et al., 2013; Van Leeuwen et al., 2022). Estimated marginal means were calculated for the two FIA categories across the three viewing conditions. A control analysis found no main effects for age or sex, so the presented results were averaged across all research participants in the study (*N* = 37). Total number of observations: 12,617. The bold values indicate a statistically significant result, defined as a p value smaller than 0.05.

Model 2 (Table 4) found strong evidence for a main effect of “FIA subcategory” and for an interaction between “Viewing condition” and “FIA subcategory,” suggesting that the audio stimuli had different effects on the visual attention for social cues in the artworks and complex images in the “Contextual information” and “External perspective” (VTS) viewing conditions (*p* < 0.001, VS-MPR = 5.382 × 10^+37^). In both the “Contextual information” and “External perspective” (VTS) viewing condition, audio references to Animate image elements led to significantly more visual attention for these specific elements, with estimated average dwell times of 2,579 ms ± 212 ms and 2,454 ms ± 219 ms, respectively. In comparison, the estimated average dwell times on Animate image elements without audio references were 962 ms ± 212 (SE) in the Contextual information viewing condition and 1,665 ms ± 209 (SE) in the External perspective (VTS) viewing condition. However, only in the External perspective (VTS) viewing condition did, audio references to Inanimate image elements also lead to significantly more visual attention for these specific elements, with an estimated average dwell time of 1,805 ms ± 219 ms (SE), compared to an estimated average dwell time of 592 ms ± 208 ms (SE) on Inanimate image elements without audio references. In contrast, in the Contextual information viewing condition, audio references to Inanimate image elements did not lead to more visual attention to these elements. The estimated average dwell times for Inanimate image elements with or without audio markers in the Contextual information viewing condition were very similar: 658 ms ± 225 ms (SE) vs. 695 ms ± 208 ms (SE), respectively.

**Table 4:** Linear mixed model (Table 2) was fitted to the average dwell times on Animate and Inanimate foveal interest areas (FIAs) with and without audio markers under the two viewing conditions that involved audio stimuli that referenced visual elements in the artworks and complex images: Contextual information and External perspective.

**Model 2: Effects of audio stimuli and social cues on gaze patterns across visual artworks and complex images in healthy adults**						
**Effect**			**df**	**ChiSq**	***p***	**VS-MPR** ^*^
Viewing condition			1, 17.96	1.911	0.184	1.182
Foveal interest area (FIA) subcategory			3, 10114.39	467.507	**< 0.001**	**3.236** **×10**^**+280**^
Viewing condition * FIA subcategory			3, 10114.39	63.797	**< 0.001**	**5.382** **×10**^**+37**^
**Fixed effects estimates**						
**Term**	**Estimate**	**SE**	**df**	***t***	***p***	**VS-MPR** ^*^
(Intercept)	1426.346	146.740	17.960	9.720	< 0.001	1.445 × 10^+6^
**Reference viewing condition: External perspective**						
Viewing condition: Contextual information	−202.873	146.740	17.960	−1.383	0.184	1.182
**Reference FIA subcategory: inanimate**						
FIA subcategory: animate	−112.519	31.893	10112.425	−3.528	**< 0.001**	**112.542**
FIA subcategory: animate audio marker	1090.315	39.376	10113.190	27.690	**< 0.001**	**1.013 × 10** ^**+159**^
FIA subcategory: inanimate audio marker	−195.003	48.323	10103.286	−4.035	**< 0.001**	**682.866**
**Interaction effects**						
Animate FIAs * Contextual information viewing condition	−148.690	31.893	10112.425	−4.662	**< 0.001**	**9168.838**
Animate audio marker FIAs * Contextual information viewing condition	265.278	39.376	10113.190	6.737	**< 0.001**	**8.710** **×10**^**+8**^
Inanimate audio marker FIAs * Contextual information viewing condition	−148.690	31.893	10112.425	−4.662	**< .001**	**9168.838**
**Estimated marginal means**						
						*95% CI*
**Viewing condition**		**Foveal interest area subcategory**	**Estimate**	**SE**	**Lower**	**Upper**
Contextual information		Animate	962.264	212.045	546.662	1377.865
External perspective		Animate	1665.390	209.406	1254.962	2075.818
Contextual information		Inanimate	694.569	207.897	287.099	1102.038
External perspective		Inanimate	592.538	207.821	185.216	999.860
Contextual information		Animate audio marker	2579.066	211.888	2163.774	2994.359
External perspective		Animate audio marker	2454.256	219.165	2024.700	2883.813
Contextual information		Inanimate audio marker	657.992	225.433	216.152	1099.833
External perspective		Inanimate audio marker	1804.694	218.711	1376.029	2233.359

Model terms tested with the Satterthwaite test method.

The following variable is used as a random effects grouping factor: “Image.”

Type III sum of squares.

The intercept corresponds to the (unweighted) grand mean; for each factor with k levels, k - 1 parameters are estimated with sum contrast coding. Consequently, the estimates cannot be directly mapped to factor levels. The estimated marginal means below provide estimates for each factor level/design cell and their differences.

^*^Vovk-Sellke maximum p-ratio: Based on a two-sided p-value, the maximum possible odds in favor of H1 over H0 equal 1/(-e p log(p)) for *p* ≤ 0.37 (Sellke et al., 2001).

Fixed effects were calculated for “viewing condition” with 2 levels (Contextual information and External perspective) and “FIA subcategory” with 4 levels (Animate, Inanimate, Animate audio marker, and Inanimate audio marker), with “Image” (*N* = 20) selected as a random effects grouping factor. The LMM random-effect structures for “Image” included only the intercept as there was not enough data to estimate the random slopes for the FIA subcategories with audio markers. All random slopes involving “Viewing condition” were automatically removed from the model by JASP as the factor “Viewing condition” did not vary within the levels of random effects grouping factor “Image.” The dwell-time data that were included in the analysis were recorded between 750 and 20,000 ms after image presentation. Estimated marginal means were calculated for the four FIA subcategories across the two Viewing conditions. A control analysis found no main effects for age or sex, so the presented results were averaged across all research participants in the study (*N* = 37). Total number of observations: 10,138. The bold values indicate a statistically significant result, defined as a p value smaller than 0.05.

While Models 1 and 2 found strong evidence for significant differences in gaze patterns across the three viewing conditions, Model 3 (Table 5) found no effect of viewing condition on the personal resonance with the artworks and complex images when taking the random effects of the image selection into account. A rating of 1 indicated that the participant felt a strong resonance with the artwork or complex image, a rating of 3 indicated a neutral feeling and a rating of 5 indicated the participant felt very little to no resonance with the artwork or complex image. The estimated average resonance rating in the Contextual information viewing condition was 2.6 ± 0.16 (SE), in the External perspective (VTS) viewing condition 2.7 ± 0.16 (SE), and in the Internal perspective (VTS) viewing condition 2.7 ± 0.16 (SE). These results suggest that the artworks and complex images that were selected as stimuli in this study resonated somewhat with the participants, but not particularly strongly, and the different viewing conditions had no influence on this.

**Table 5:** Generalized linear mixed model (Model 3) was fitted to the (ordinal) personal resonance ratings of the visual artworks and complex images (*N* = 30), with “viewing condition” as a fixed effect and “Image” as a random effects grouping factor.

**Model 3: Effects of different viewing conditions on personal resonance with visual artworks and complex images in healthy adults**				
**Effect**	**Df**	**ChiSq**	***p***	**VS-MPR** ^*^
Viewing condition	2	0.401	0.818	1.000
**Estimated marginal means**				
				*95% CI*
Viewing condition	Estimate	SE	Lower	Upper
Contextual information	2.586	0.160	2.272	2.900
External perspective	2.720	0.160	2.406	3.034
Internal perspective	2.678	0.160	2.364	2.992

Two observations were removed due to missing values.

Generalized linear mixed model with Gaussian family and identity link function.

Model terms tested with likelihood ratio tests Method.

The following variable is used as a random effects grouping factor: “Image” (*N* = 30).

Type III sum of squares.

^*^Vovk–Sellke maximum p-ratio: Based on a two-sided *p*-value, the maximum possible odds in favor of H1 over H0 equal 1/(-e p log(p)) for *p* ≤ 0.37 (Sellke et al., 2001).

The estimated marginal means below provide estimates for each factor level/design cell and their differences.

No effect of viewing condition on resonance ratings was found, and a control analysis showed that there were neither effects of age nor sex; hence, the presented results were averaged over all research participants (*N* = 36). Total observations: 1,078. A rating of 1 indicated a strong resonance with the artwork or complex image, a rating of 3 indicated a neutral response and a rating of 5 indicated very little to no resonance with the artwork or complex image. The estimated marginal means suggest that participants on average felt some resonance with the artworks and complex images that were selected as stimuli in this study but not particularly strongly. The different viewing conditions had no influence on this.

One participant from the young adults cohort was excluded from the analysis because their resonance ratings were much lower than average across all three viewing conditions (>2 std dev), possibly suggestive of an indiscriminate low engagement with and/or dislike of the image selection.

**Figure 2:**
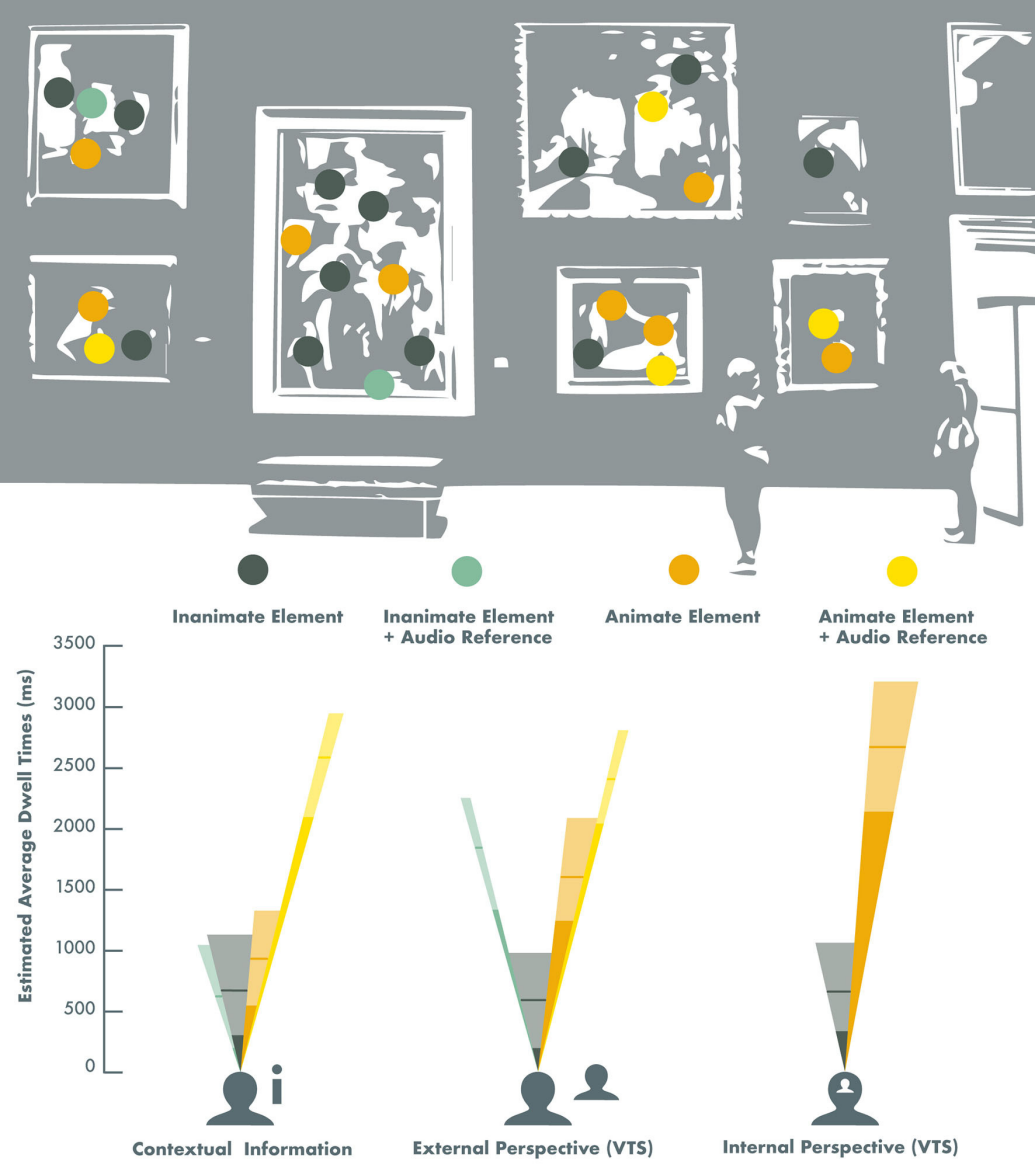
Effects of different viewing conditions and auditory input on gaze patterns across visual artworks and complex imagery in healthy adults. The height of the different colored dwell-time beams represents the estimated average gaze dwell time on Animate (orange) and Inanimate (dark green) image elements, and the subtended angles of the visual field beams represent the proportional representation of these image elements across the image selection. The yellow beams represent the estimated average gaze dwell times (ms) on Animate elements that were specifically referred to in a pre-recorded audio recording that was played simultaneously with the image presentation, and the light green beams represent the estimated average gaze dwell times (ms) on Inanimate image elements with audio references (*Note*: this only concerned the Contextual information and the External perspective viewing conditions). The lighter sections of the beams correspond with the 95% confidence intervals, and the horizontal lines represent the estimated value in the models. The estimated gaze dwell times for the Internal perspective (VTS) viewing condition were derived from Model 1 (Table 3), and the estimated gaze dwell times for the Contextual information and the External perspective viewing conditions were derived from Model 2 (Table 4).

Figure 2 illustrates the effects of different viewing conditions and auditory input on gaze patterns and personal resonance with visual artworks and complex imagery.

Supplementary Table 4 shows the descriptive statistics of the measured gaze patterns across visual artworks and complex images during 20 s viewings under different viewing conditions in healthy adults.

## Discussion

In this study, we applied the theoretical frameworks of the social and artistic brain connectomes to an eye-tracking paradigm with the aim to elucidate how different viewing conditions and social cues influence gaze patterns and personal resonance with artworks and complex imagery in healthy young and older adults. We compared two viewing conditions that encourage personal or social perspective taking—modeled on the well-known Visual Thinking Strategies (VTS) method—to a viewing condition during which only contextual information about the image was provided. Our findings confirmed our hypothesis that social cues (Animate vs. Inanimate elements) in artworks and complex imagery influence gaze patterns. We found that a participant's age or sex had no effect on this, but viewing condition did have a strong effect. Viewing conditions that encourage personal or social perspective taking (VTS) directed the gaze more toward highly salient social cues (Animate elements) in artworks and complex imagery, compared to when only contextual information was provided. We furthermore found that audio cues also directed visual attention, whereby listening to a personal reflection by another person (VTS) had a stronger effect than contextual information. However, we found no effect of viewing condition on the personal resonance with the artworks and complex images, when taking the random effects of the image selection into account. Across all three viewing conditions in this study, participants resonated somewhat with the images from the Wellcome Collection but not particularly strongly. This could perhaps partly be explained by the nature of the Wellcome Collection, which is mostly historical and medically orientated, but it is also a reminder that resonating strongly with an artwork or image does not happen frequently and is furthermore highly personal, which Vessel et al. (2013) have previously demonstrated.

For the purpose of this study, we deliberately did not tailor the image selection to the research participants, but it should be noted that the VTS method explicitly recommends taking contextual factors, cognitive capacity, and personal interests into account in the artwork/image selection to optimize audience engagement (Yenawine, 2003).

Our study provides a neurobiological grounding of the VTS method in the social brain by demonstrating that personal and social perspective taking have distinct effects on both self-guided and other-directed visual attention. It is important to emphasize that gaze or visual attention is not a pedagogical “outcome” in itself as VTS is likely to exert its effects via a sequence of cognitive operations. However, our reductive paradigm here has allowed us to identify a facilitatory physiological mechanism for orienting or priming the cognitive processes that mediate the behavioral effects of VTS. As outlined in the Introduction, the artistic brain connectome provides a neural “roadmap” by which attentional shifts in perceptual processing can channel the flow of information about artworks through interacting social brain networks. Our findings suggest that exploring artworks or complex images with the VTS method promotes stronger engagement of the social brain networks, which can ultimately influence higher cognitive operations and the programming of output behaviors. Beyond art, gaze recordings have been shown to mirror neural mechanisms engaged in a variety of complex perceptual and cognitive processes, in humans and other primate species (Corbetta and Shulman, 2002; Henderson, 2003; Dalmaso, 2022; Lewis and Krupenye, 2022). The present findings complement behavioral studies which have found that VTS promotes personal and social engagement with artworks and complex imagery (Housen and Yenawine, 2000-2001; Housen, 2002, 2007; Yenawine, 2003, 2013; Naghshineh et al., 2008; Miller et al., 2013; Miller and Yenawine, 2014; Van Leeuwen et al., 2021; Ferrara et al., 2022).

This study has several limitations that suggest clear directions for future work. Our paradigm here was deliberately reductive, presenting artworks in reproduction and isolation in a lab environment; moreover, the study included a relatively limited emotional range of artworks, with no prior personal relevance. We do not argue that our experimental setup captures the richness of encountering artworks in the world at large, or the experience of viewing art with other people: These factors are likely to heavily influence art-viewing behaviors (Estrada-Gonzalez et al., 2020).

The superior ability of VTS to enhance visual attention for social cues provides empirical support for the core premise of social constructivism that learning and cognitive development take place in a social context and depend fundamentally on interactions with others (Bruner, 1960, 1986; Vygotsky, 1962). This potentially has real-world implications, not only for (art) education but also for art-based therapeutic applications, especially in patient populations with diminished mental abilities (e.g., people living with dementia). Engaging people with art from a personal and social perspective using VTS is likely to be more beneficial than taking a top-down didactic approach. However, establishing these potential benefits will require future studies building on our paradigm that close the considerable gap that separates the laboratory from the experience of encountering art in the real world. We hope that future studies will exploit this potential to adapt our paradigm to real-world viewing conditions, including viewing art in the company of other people. We further envisage that Eye-tracking and other physiological tools could become part of the psychometric inventory used to assess the behavioral, pedagogical, and clinical outcomes of VTS and other interventions that employ artworks to enhance personal wellbeing.

In conclusion, this study offers a strong *prima facie* case for social brain engagement by the VTS method. Further research is needed to delineate the neural correlates of our findings, as well as their application in real-world environments.

## Data availability statement

The datasets generated and/or analyzed during the current study are not publicly available due to the stipulation of the institutional ethics approvals covering consent and data collection, but are available from the corresponding author on reasonable request.

## Ethics statement

The studies involving humans were approved by University College London Research Ethics Committee (8545/002: Created Out of Mind) and the UCL Queen Square Research Ethics Committee (17/LO/0099). The studies were conducted in accordance with the local legislation and institutional requirements. The participants provided their written informed consent to participate in this study.

## Author contributions

JL: conceptualization, methodology, investigation, resources, data curation, formal analysis, writing—original draft, writing—reviewing and editing, visualization, and project administration. SC and JW: conceptualization, methodology, supervision, and writing—reviewing and editing. All authors contributed to the article and approved the submitted version.
