# Supplementary material for: Thinking eyes: visual thinking strategies and the social brain
Source: Front Psychol. 2023 Sep 27;14:1222608. doi: 10.3389/fpsyg.2023.1222608 (PMC10565500; doi:10.3389/fpsyg.2023.1222608)
Supplement: Supplementary file 1 [file Data_Sheet_1.docx]

**Thinking Eyes: Visual Thinking Strategies and the Social Brain, by Janneke EP van Leeuwen, Sebastian J Crutch, and Jason D. Warren**

**Supplementary Materials**

**Supplementary Figures**

**
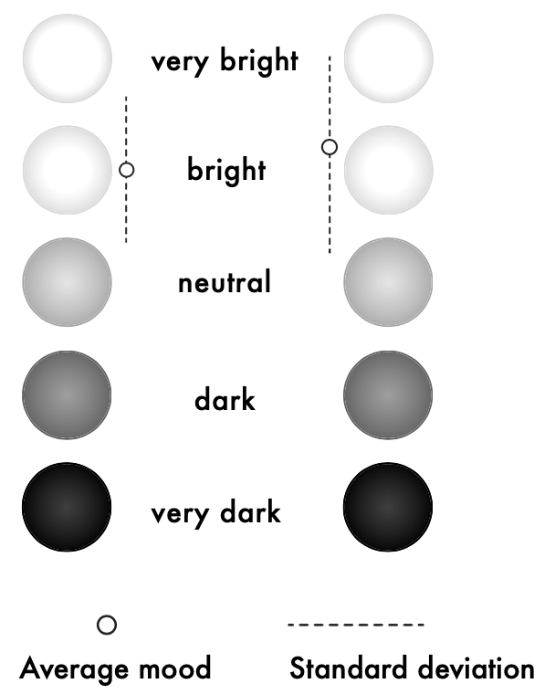
**

**Supplementary Figure 1** Self-reported mood of participants on the novel Mood Shade Scale (Van Leeuwen, 2020, CC BY 4.0). The left side of the figure shows the average mood of young adults on the day they took part in the study. The right side of the figure shows the average mood of older adults on the day they took part in the study. The average mood of participants was bright, and there was no statistically significant difference between Young and Older Adults.

**
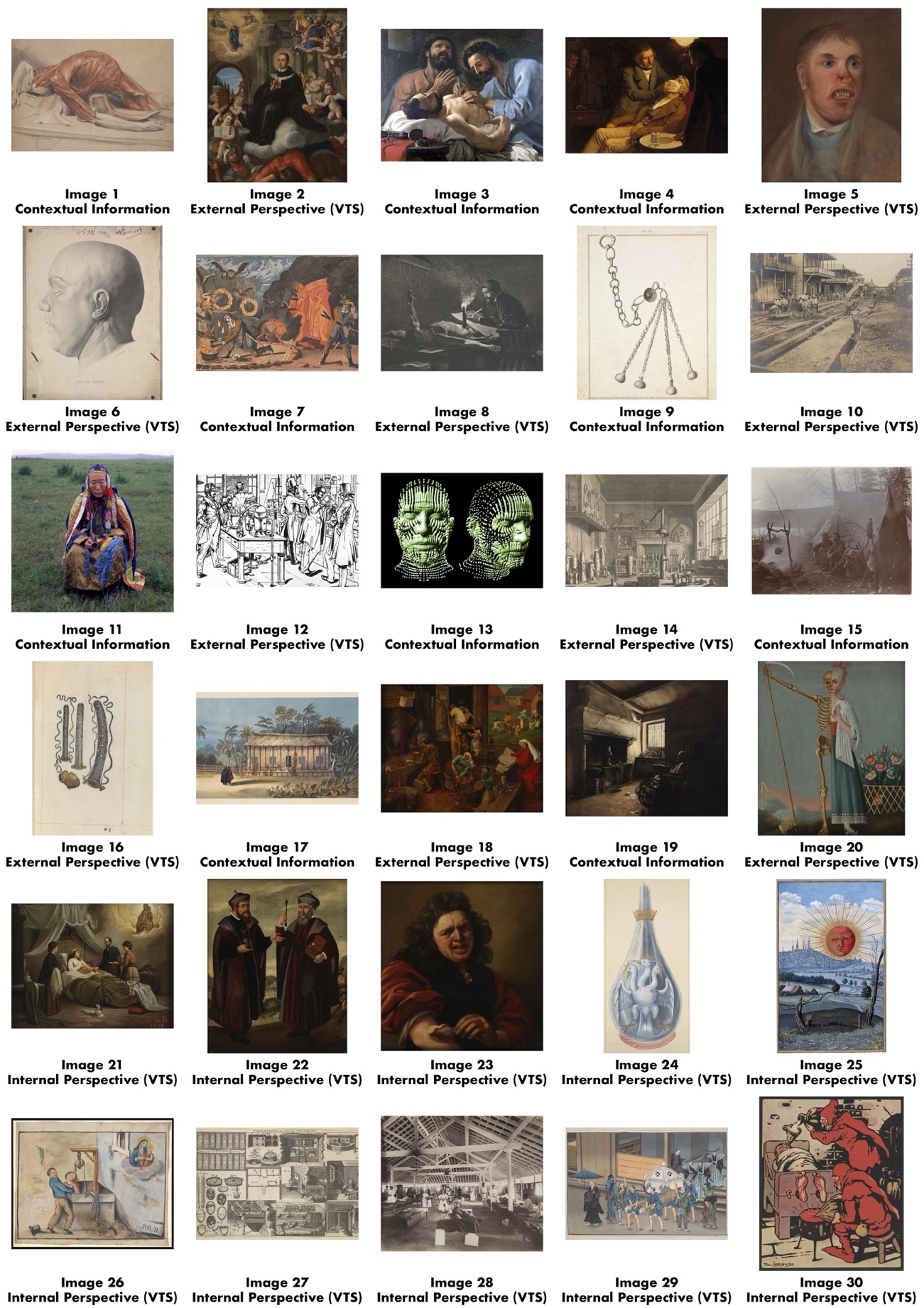
**

**Supplementary Figure 2:** Overview of the Experimental Stimuli Selection from the Wellcome Collection, London, UK. All visual artworks and complex imagery used as experimental stimuli in this study were selected from the open-access online library of the Wellcome Collection, London. See Supplementary Table 1 for a full list of image attributions and publication licenses.

**
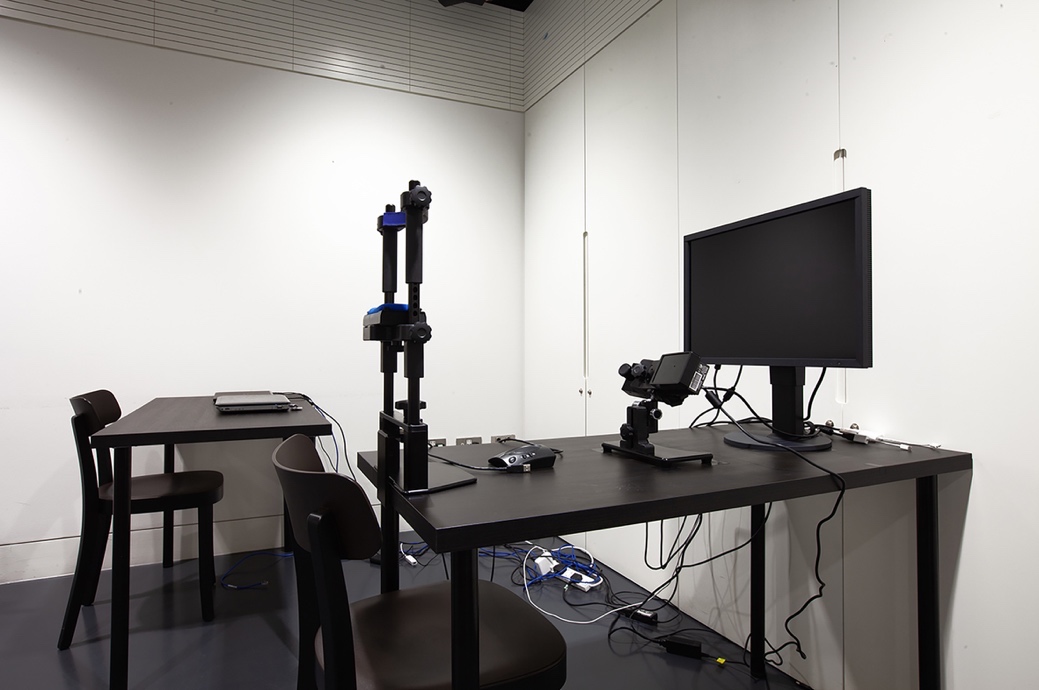
**

**Supplementary Figure 3**: Set-up of the eye tracking experiments. During the experiments the ambient light was switched off and participants were given time to adjust to the darkness of the room before the start of the first trial.

**
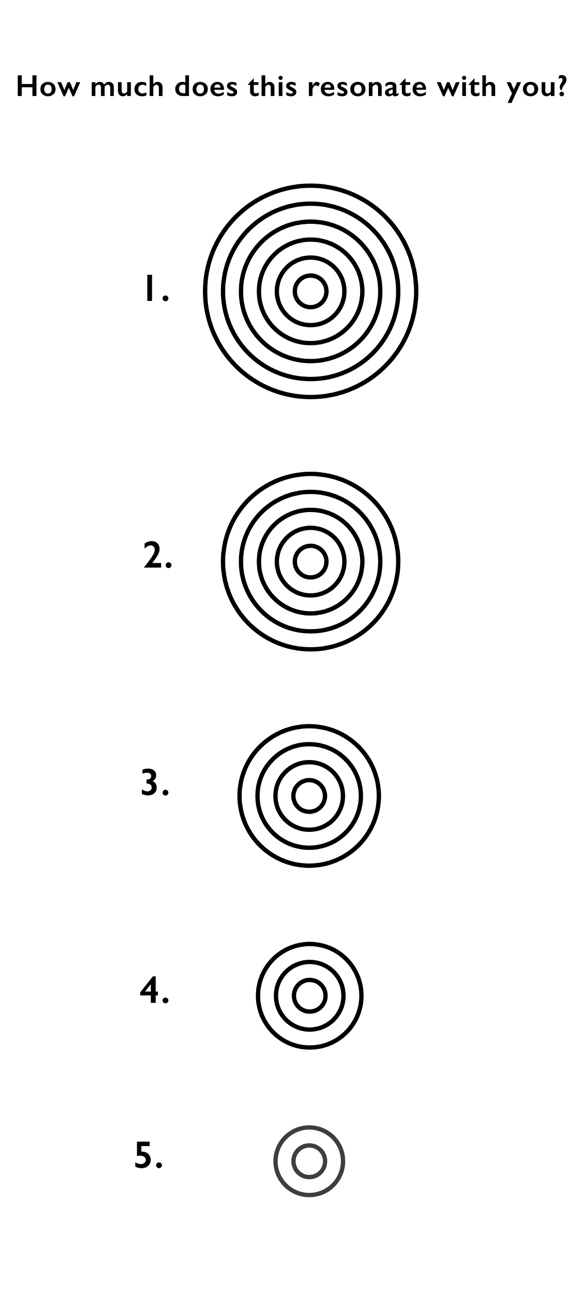
**

**Supplementary Figure 4:** The ‘Resonance Radiance Scale’ a novel visual rating scale that was designed specifically for this study to measure the study participants’ personal resonance with visual artworks and complex imagery on a scale of 1 (strongly resonates) to 5 (very little to no resonance).

**Supplementary Tables**

| **Image Attributions of Experimental Stimuli Selected from the Wellcome Collection Library Catalogue, London, UK** | | |
| --- | --- | --- |
| *Image* | *Attribution* | *Licence* |
| 1 | [Chalk drawing of a flayed corpse by Charles Landseer, c.1815.](https://wellcomecollection.org/works/uepzw7md) | Public Domain Mark |
| 2 | [Apotheosis of an Ecclesiastic. Oil painting. Italian, date unknown.](https://wellcomecollection.org/works/h5uwdhyz) | Attribution 4.0 International (CC BY 4.0) |
| 3 | [Saint Cosmas and Saint Damian Dressing a Chest Wound. Antonie de Favray, 1748.](https://wellcomecollection.org/works/eazd6hes) | Attribution-Non-Commercial 4.0 International (CC BY-NC 4.0) |
| 4 | [The First Use of Ether as an Anaesthetic in Dental Surgery. Oil on canvas. Ernest Board, c. 1912.](https://wellcomecollection.org/works/t99vetuz#?asi=0&ai=0) | Attribution 4.0 International (CC BY 4.0) |
| 5 | [A Young Man, Mr J Kay, afflicted with a disease which has eaten away part of his face. Oil painting, ca. 1820.](https://wellcomecollection.org/works/gzgzm2sf#?asi=0&ai=0) | Attribution 4.0 International (CC BY 4.0) |
| 6 | [The death mask of William Palmer, the poisoner. Lithograph after M. Krantz, c. 1860.](https://wellcomecollection.org/works/xqyqq7p7) | Attribution 4.0 International (CC BY 4.0) |
| 7 | [Coloured engraving with gouache of a man conducting magic rites and a hunter cowering in terror, nineteenth century.](file:///C:\Users\jannekevanleeuwen\Janneke\02%20Professional\02%20The%20Thinking%20Eye\01%20Research%20&%20Development\01%20Research\03%20Academic%20Papers\2023\Thinking%20Eyes_VTS%20and%20the%20Social%20Brain\Attribution%204.0%20International%20(CC%20BY%204.0)) | Attribution 4.0 International (CC BY 4.0) |
| 8 | [Michelangelo drawing from an anatomized cadaver. Photogravure after M. J. A. Mercié. Mercié, Antonin, 1845-1916.](https://wellcomecollection.org/works/fj2x6zuf#?asi=0&ai=0) | Attribution 4.0 International (CC BY 4.0) |
| 9 | [Engraving of an instrument of torture used against early Christians James Basire after Robert Stothard, 1827.](https://wellcomecollection.org/works/p3fxasfm) | Attribution 4.0 International (CC BY 4.0) |
| 10 | [Photograph of sewers built for the removal of rainwater during construction of the Panama Canal. Henry Wellcome, 1910.](https://wellcomecollection.org/works/zq2jcp6d#?asi=0&ai=0) | Attribution 4.0 International (CC BY 4.0) |
| 11 | [Photograph of a Shaman healer in Dorgut, Mongolia by Mark de Fraeye.](https://wellcomecollection.org/works/tf6rp8zv) | Attribution-Non-Commercial-No Derivatives 4.0 International (CC BY-NC-ND 4.0) |
| 12 | [Leibig's Laboratory at Giessen Drawing 19th Century By: William Trautschold. From: Berolzheimer Series of Alchemical and Historical Reproductions.](https://wellcomecollection.org/works/vbyhp8fa) | Attribution 4.0 International (CC BY 4.0) |
| 13 | [Constructing a human face. Spooky Pooka, 2005.](https://wellcomecollection.org/works/xhm96e5a) | Attribution-Non-commercial-No Derivatives 4.0 International (CC BY-NC-ND 4.0) |
| 14 | [Engraving of an alchemical laboratory from Philosophical Commerce of Arts, William Lewis, 1765.](https://wellcomecollection.org/works/a27suwqc) | Attribution 4.0 International (CC BY 4.0) |
| 15 | [Photograph of Henry Wellcome's camp in Maine, 1886.](https://wellcomecollection.org/works/wgacsbp2) | Attribution 4.0 International (CC BY 4.0) |
| 16 | [Instrumenta Chyrurgiae et Icones Anathomicae, Ambroise Paré 1564.](https://wellcomecollection.org/works/gea92cjg/images?id=zg824gqf) | Public Domain Mark |
| 17 | [Coloured etching of a bamboo cottage built for an Englishman, by Charles Empson, 1836.](https://wellcomecollection.org/works/wwx79anw) | Attribution 4.0 International (CC BY 4.0) |
| 18 | [An Alchemist. Oil painting. After Pieter Bruegel the Elder.](https://wellcomecollection.org/works/jyrck5qt) | Attribution 4.0 International (CC BY 4.0) |
| 19 | [An Alchemist. Oil painting. Eugène Lomont, 1890.](https://wellcomecollection.org/works/fxamv69x) | Attribution 4.0 International (CC BY 4.0) |
| 20 | [A woman divided into two, representing life and death. Oil painting.](https://wellcomecollection.org/works/qwfwb7bm) | Attribution 4.0 International (CC BY 4.0) |
| 21 | [A Woman in Bed in a Sick Room. Oil painting. R Pistoni, 1872.](https://wellcomecollection.org/works/adgwyu7v) | Public Domain Mark |
| 22 | [Saint Cosmas and Saint Damian in a Landscape. Oil on canvas Spain, 17th century.](https://wellcomecollection.org/works/wtkum788) | Attribution 4.0 International (CC BY 4.0) |
| 23 | [The Sense of Touch. Oil painting. Lucas Franchoys the Younger, 17th century.](https://wellcomecollection.org/works/epy5sv3z) | Attribution 4.0 International (CC BY 4.0) |
| 24 | [Watercolour of a three-headed eagle, representing the sublimation of mercury three times, after Salomon Trismosin's sixteenth-century alchemical manuscript, Splendor Solis.](https://wellcomecollection.org/works/nq5v5cb5) | Attribution 4.0 International (CC BY 4.0) |
| 25 | [Watercolour representing the culmination of the alchemical Great Work or the star of hope that inspires the alchemist, after Salomon Trismosin's sixteenth-century alchemical manuscript, Splendor Solis.](https://wellcomecollection.org/works/qtnzn2mz) | Attribution-Non-Commercial 4.0 International (CC BY-NC 4.0) |
| 26 | [Votive watercolour of a woman, who is being rescued from a well, praying to Sansovino’s Virgin and Child.](https://wellcomecollection.org/works/t24rrugj) | Attribution 4.0 International (CC BY 4.0) |
| 27 | [Engraving of factory-farming of eggs, eighteenth century.](https://wellcomecollection.org/works/zencg9qs) | Attribution 4.0 International (CC BY 4.0) |
| 28 | [Photograph of a temporary hospital for plague victims during the Bombay plague epidemic, 1897.](https://wellcomecollection.org/works/awct3kzq) | Attribution 4.0 International (CC BY 4.0) |
| 29 | [Watercolour of a Japanese funeral cortège leaving a dead man’s house for the temple c. 1880.](https://wellcomecollection.org/works/rbdpzp6w) | Attribution 4.0 International (CC BY 4.0) |
| 30 | [Colour process print satirising Fabricus von Hilden’s view that gout could be cured by torture Daniel Thoroude de Losques, 1910.](https://wellcomecollection.org/works/kbvyc8aw) | Attribution 4.0 International (CC BY 4.0) |

**Supplementary Table 1:** Image Attributions of Experimental Stimuli Selected from the Wellcome Collection Library Catalogue, London, UK.

| *Foveal Interest Areas* | | *Contextual Information*  *Mean Dwell Time (ms)* | *External Perspective*  *Mean Dwell Time (ms)* | *Internal Perspective*  *Mean Dwell Time (ms)* |
| --- | --- | --- | --- | --- |
|  | **Animate** | **1314.395** | **1855.469** | **2240.457** |
| 1 | Animate image centre | 2248.152 | 1674.752 | 3023.853 |
| 2 | Frontal human faces | 2199.131 | 3442.714 | 3609.143 |
| 3 | Frontal animal faces | 1144.371 | N/A | 2722.423 |
| 4 | Sideways human faces | 1472.286 | 1861.899 | 2370.817 |
| 5 | Sideways animal faces | 1425.536 | N/A | 885.929 |
| 6 | Human hand actions | 583.750 | 917.746 | 2507.714 |
| 7 | Animal hand actions | N/A | N/A | N/A |
| 8 | Human body elements | 815.724 | 1380.234 | 1445.702 |
| 9 | Animal body elements | 626.210 | N/A | 1358.076 |
|  | **Inanimate** | **1276.297** | **759.675** | **561.381** |
| 10 | Inanimate image centre | 4049.093 | 919.307 | 589.819 |
| 11 | Text elements | 344.514 | 1523.036 | 354.204 |
| 12 | Human-made objects | 952.046 | 696.307 | 613.846 |
| 13 | Built environment elements | 612.107 | 580.084 | 760.634 |
| 14 | Natural elements | 423.723 | 508.500 | 488.403 |
| 15 | Number elements | N/A | 330.814 | N/A |

**Supplementary Table 2:** Overview of the taxonomy of the Foveal Interest Areas (FIAs) that were used in this study, which were derived from the functional profiles of the core hubs in the Perception Network of the social brain connectome (Alcala-Lopez et al., 2017). See further detailed under the section Gaze Patterns in Supplementary Materials. Average gaze dwell times (ms) — including audio cues — on the separate Foveal Interest Area subcategories within the Animate and Inanimate domains are listed in columns for each of the three different viewing conditions. Not every subcategory was present in each viewing condition across the image selection, which is indicated with N/A.

*Note.*  Results were averaged over the levels of: Cohort (Young and Older Adults) and Sex (Female and Male).

*Note*. In the statistical analyses the average gaze dwell times (ms) on the individual FIAs in each image were averaged across the Animate and Inanimate categories and split in With/Without Audio Marker subcategories.

| *Viewing Condition* | *Image* | *Audio Transcript* |
| --- | --- | --- |
| Contextual Information | 1 | Chalk drawing of a flayed corpse, Charles Landseer, 1815. |
| Contextual Information | 3 | Saint Cosmas and Saint Damian Dressing A Chest Wound. By Antoine de Favray, 1748. |
| Contextual Information | 4 | The first use of ether as an anaesthetic in dental surgery. Oil on canvas, by Ernest Board, 1912. |
| Contextual Information | 7 | Coloured engraving with gouache, of a man conducting magic rites and a hunter cowering in terror, 19th Century. |
| Contextual Information | 9 | Engraving of an instrument of torture used against Early Christians. By James Baseer, after Robert Stothard, 1827. |
| Contextual Information | 11 | Photograph of a Shaman Healer in Dakgoot, Mongolia. By Mark de Frevay. |
| Contextual Information | 13 | Computer generated image of a man's face, by Spooky Pooka. 2005 |
| Contextual Information | 15 | Photograph of Henry Wellcome's Camp in Maine, 1886. |
| Contextual Information | 17 | Coloured etching of a bamboo cottage, built for an English man. By Charles Epson, 1836. |
| Contextual Information | 19 | An Alchemist, oil on canvas by Eugene Lamont, 1890. |
| External Perspective | 2 | It's quite er, busy image, erm, I'm being drawn towards the, the, kind of door at the back that seems to be leading out to, to a forest of some sort. |
| External Perspective | 5 | So, I'm first struck by the somewhat grotesque, kind of, erm, cut on the man's nose. That looks fairly painful, and then, weirdly, I look at the background- |
| External Perspective | 6 | Suddenly my eyes flash up to the top and the scribbles, and the, and the numbers. Erm, but the man looks quite content and somewhat at peace. |
| External Perspective | 8 | So it's quite a, somewhat, eerie and shadowy image. First of all, I'm drawn to the, erm, fire in the middle, that's lighting up the man's face and seems like he's coming out of the other man's body. |
| External Perspective | 10 | So, this looks like a photograph that's- what I'm immediately drawn to, it's the fact that everybody in seems, well, not everybody, but many people are staring at the photographer. |
| External Perspective | 12 | For some reason, I'm drawn into one of the middle guys whose eye is, kind of, poking out in a slightly odd direction almost. And it feels like you're being stared at. |
| External Perspective | 14 | I feel like this is some sort of guitar or musical instrument, in the left-hand corner, erm, and it almost takes a second to, kind of, once you focus in on each object, to, erm, to figure out what, what its place is in the room. |
| External Perspective | 16 | Weirdly, I'm drawn to the, kind of, weird ball of, er, I'm not sure what it is? Something fluffy or spikey or- |
| External Perspective | 18 | For some reason I'm drawn to the face of the, of the man in the red robe on the right, and the face of the man in the green robe on the floor. And they both look to have, almost, exact same facial expression. |
| External Perspective | 20 | Mmm, weirdly looking downwards the side of the skeleton photo, and the book almost looks like its got a pair of legs. And then at the candle that's broken in half. |

**Supplementary Table 3:** Transcripts of the audio stimuli that were played to the research participants during the presentation of the artworks and complex images in the Contextual Information and External Perspective (VTS) viewing conditions. All audio stimuli in both viewing conditions were recordings of the same female voice, a volunteer with native British nationality and an excellent command of Standard British English. The audio stimuli in the Contextual Information viewing condition consisted of the catalogue information about that image that was provided by the Wellcome Collection’s online archive. The audio stimuli in the External Perspective viewing conditions were snippets from the volunteer’s unscripted responses to the VTS question: ‘What is going on in this picture?’. The audio files from the External Perspective viewing condition only included the first couple of sentences of the personal reflection, to ensure the cognitive load of the audio stimuli was not much larger compared to the Contextual Information viewing condition.

*Note*. In the Internal Perspective viewing condition, the same audio stimulus was played during every image trial and consisted of the VTS prompting question: ‘What is going on in this picture?’.

| **Gaze patterns on visual artworks and complex imagery across different viewing conditions in healthy adults** | | | | | | | |
| --- | --- | --- | --- | --- | --- | --- | --- |
| *Viewing Condition* | *Foveal Interest Area (FIA) Category* | *Total number of FIA’s* | *N* | *Mean* | *SD* | *SE* | *Coefficient of variation* |
| Contextual Information | Animate | 55 | 35 | 773.493 | 148.636 | 25.124 | 0.192 |
|  | Animate Audio Marker | 29 | 35 | 1799.218 | 413.898 | 69.961 | 0.230 |
|  | Inanimate | 77 | 35 | 739.343 | 147.935 | 25.006 | 0.200 |
|  | Inanimate Audio Marker | 15 | 35 | 902.846 | 307.281 | 51.940 | 0.340 |
| External Perspective (VTS) | Animate | 54 | 35 | 1441.447 | 170.650 | 28.845 | 0.118 |
|  | Animate Audio Marker | 11 | 35 | 2240.049 | 684.187 | 115.649 | 0.305 |
|  | Inanimate | 86 | 35 | 538.479 | 80.679 | 13.637 | 0.150 |
|  | Inanimate Audio Marker | 10 | 35 | 1847.091 | 785.492 | 132.772 | 0.425 |
| Internal Perspective (VTS) | Animate | 50 | 35 | 2132.652 | 283.432 | 47.909 | 0.133 |
|  | Inanimate | 75 | 35 | 608.827 | 102.543 | 17.333 | 0.168 |

**Supplementary Table 4**: Descriptive statistics of the average (mean) gaze dwell times (ms) on Animate and Inanimate Foveal Interest Areas (FIAs), with and without Audio Markers, in artworks and complex imagery under different viewing conditions in healthy adults.

*Note.*  Results were averaged over the levels of: Cohort (Young and Older Adults) and Sex (Female and Male) *Note.*   Mean gaze dwell times were calculated over the 750-20000 ms presentation window
